# Ameliorative effect of montelukast against STZ induced diabetic nephropathy: targeting HMGB1, TLR4, NF-κB, NLRP3 inflammasome, and autophagy pathways

**DOI:** 10.1007/s10787-023-01301-1

**Published:** 2023-07-27

**Authors:** Ahmed M. Awad, Sally L. Elshaer, Rajashekhar Gangaraju, Rania R. Abdelaziz, Manar A. Nader

**Affiliations:** 1 Department of Pharmacology and Toxicology, Faculty of Pharmacy, Mansoura University, Mansoura, 35516 Egypt; 2 Department of Ophthalmology, University of Tennessee Health Science Center, Memphis, TN 38163 USA; 3 Department of Anatomy & Neurobiology, University of Tennessee Health Science Center, Memphis, TN 38163 USA

**Keywords:** Montelukast, STZ, Renoprotection, HMGB1, NF-κB, NLRP3, Autophagy

## Abstract

Diabetic nephropathy (DN) is reported as one of the most serious microvascular diabetic complications and the trigger of end-stage renal disease (ESRD), underscoring the concern of any therapeutic intervention directed at ameliorating the development and progression of DN. The current study explored the renoprotective impact of montelukast (Mon) against streptozotocin (STZ)-induced DN in rats compared to a standard anti-hyperglycemic insulin (Ins) treatment. Diabetes was induced by a single dose of STZ (55 mg/kg). Diabetic rats were treated with Mon (10 and 20 mg/kg, oral gavage) for eight weeks. Mon administration for 8 weeks after induction of diabetes conferred significant dose-dependent renoprotection, independent of blood glucose levels (unlike Ins), as evidenced by the improvement in serum creatinine, and blood urea nitrogen (BUN), and ameliorated STZ-induced renal necrotic, inflammatory alterations, and renal fibrosis. Additionally, Mon treatment in diabetic rats significantly restored redox hemostasis as evidenced by malondialdehyde (MDA) and total antioxidant capacity (TAC) levels; significantly reduced the renal expression of high mobility group box (HMGB) 1, toll-like receptor (TLR) 4, nuclear factor kappa B (NF-κB) (in the nucleus), NOD-like receptor family pyrin domain containing (NLRP) 3, and interleukin (IL)-1β. Moreover, Mon administration ameliorated the dysregulation in autophagy as evidenced by p62 and microtubule-associated protein 1A/1B-light chain 3 (LC3)-II levels. In conclusion, the renoprotective effect of Mon is potentially associated with its modulatory effect on inflammatory cytokines, antioxidant properties, and autophagy.

**Graphical Abstract:**

Renoprotective effect of montelukast and its underlying pathway: Hyperglycemia and advanced glycation end products (AGEs) stimulate the release of high mobility group box (HMGB) 1 from necrotic and inflammatory cells. HMGB1 is considered as one of the endogenous ligands of toll-like receptor (TLR) 4, and the interaction of HMGB1 with TLR4 results in a subsequent translocation of nuclear factor kappa B (NF-κB) from the cytoplasm into the nucleus inducing an inflammatory response. NF-κB is a key mediator of the priming signal responsible for the activation of NOD-like receptor family pyrin domain containing (NLRP) 3 inflammasome by stimulating the expression of both NLRP3 and pro- interleukin (IL)-1β, which is then converted to IL-1β by to mediate inflammation. NLRP3 can induce reactive oxygen species production, while autophagy inhibits AGEs and NLRP3 accumulation. Montelukast show an inhibitory effect on HMGB1, TLR4, NF-κB, NLRP3, and IL-1β and has autophagy stimulating characteristics indicating its potential renoprotective effect.

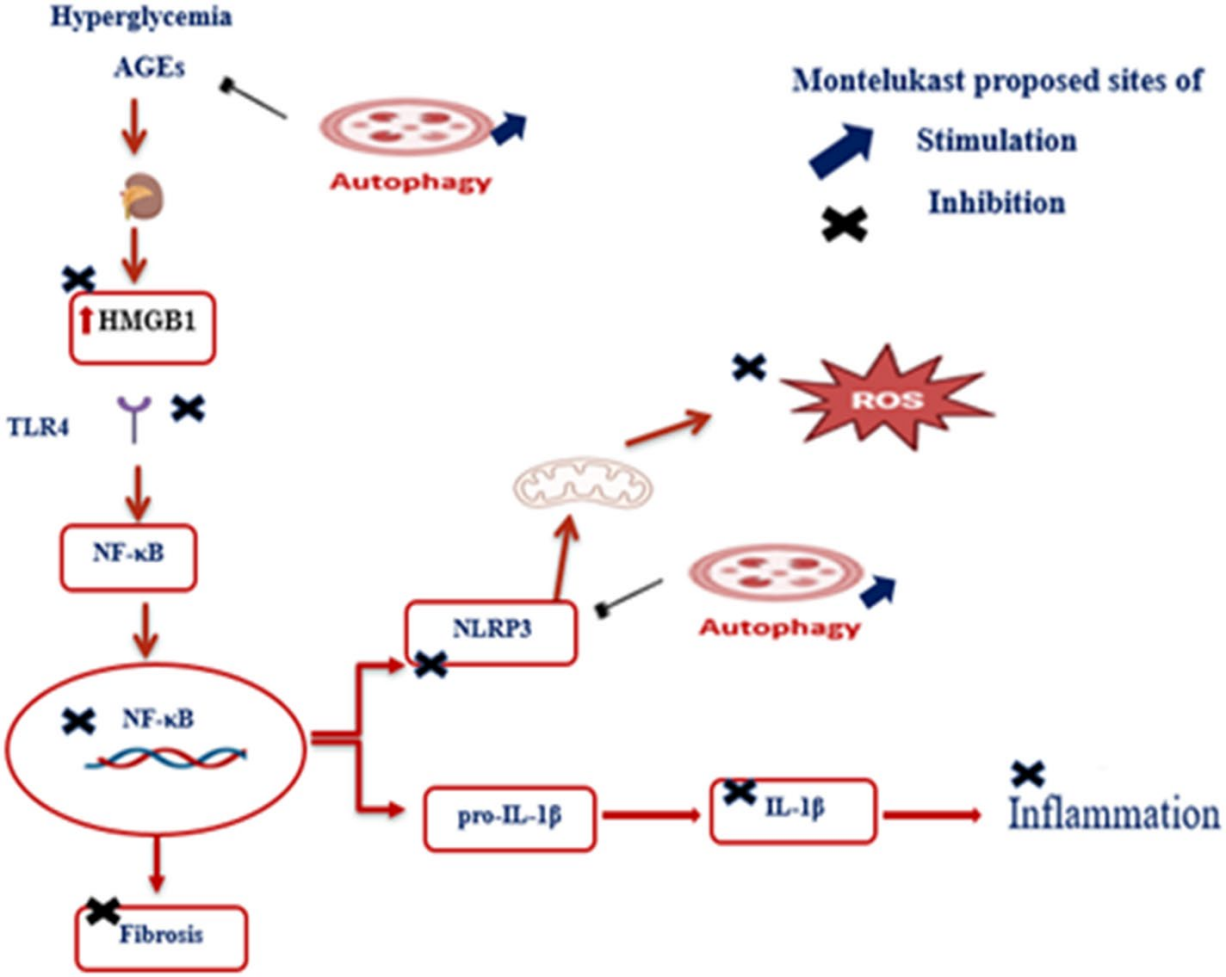

## Introduction

Diabetes and its complications significantly impact almost every age group and country (Deshpande et al. 2008). The international diabetes federation states that 537 million adults had diabetes worldwide in 2021, and this number is expected to rise to 643 and 783 million by 2030 and 2045, respectively (Sun et al. 2022). Moreover, 4.2 million deaths were attributed to diabetes in 2020 and more than 12% of the global health expenditure in the same year was allocated to deal with the disease and its complications (Shi and Hu 2014). Diabetic nephropathy (DN) is reported as one of the most serious microvascular diabetic complications and the trigger of end-stage renal disease (ESRD). It is responsible for the significant increase in both morbidity and mortality in diabetic patients (Chawla et al. 2016). The downsides of insulin (Ins) therapy, the standard treatment for diabetic complications, are the need to increase the dose, regimen complexity with time, increase in severe hypoglycemia, potential increase in mortality, and increased risk for specific cancers (Lebovitz 2011). These limitations of the chronic use of Ins underscore the concern of any therapeutic intervention directed at ameliorating the development and progression of DN.

The abnormalities in intracellular metabolism induced by diabetes, such as the production of advanced glycation end products (AGEs), cause kidney injury. AGEs accumulation in the kidney stimulates the pathogenesis of DN. For example, it stimulates the production of reactive oxygen species (ROS) and induces the production of chemokines by inflammatory cells (Ding and Choi 2015). High-mobility group box 1 (HMGB 1), one of the central chemokines produced by inflammatory cells, stimulates the release of cytokines, including interleukin (IL)-1β and IL-6 (Messmer et al. 2004) through binding to receptors for AGEs (RAGE), the first-discovered HMGB1 receptor, and Toll-like receptor (TLR)-4 leading to nuclear factor kappa B (NF-κB) activation and its translocation into the nucleus (Wu et al. 2016).

Autophagy can be defined as a conserved homeostatic cellular event. It is considered a bulk degradation process responsible for clearing and removing damaged organelles and proteins (Liu et al. 2017). The induction or stimulation of autophagy and autophagosome formation can be evidenced by the conversion of a cytosolic microtubule-associated protein 1A/1B-light chain (LC)3-I to a membrane-bound LC3-II because LC3-II is reliably associated with the formation of phagophores, both inner and outer membrane, as well as completed autophagosomes (Liang et al. 2008). So, a well-accepted indication for autophagy induction or stimulation is the high level of LC3-II or low level of p62 which is autophagic substrate degraded by autophagy (Klionsky et al. 2016). Defective autophagy was reported to play key pathogenic roles in many diseases, including DN, and it was observed in streptozotocin (STZ) induced diabetic mice, as indicated by the accumulation of p62, a substrate of autophagy-lysosomal degradation pathway (Yang et al. 2018) and low expression of LC3-II (Ma et al. 2022). Additionally, autophagy inhibits AGEs accumulation, thus encouraging AGE clearance and avoiding their renal buildup by autophagy induction may have renoprotective effects.

Montelukast (Mon) is a leukotriene receptor antagonist with high affinity and selectivity to the cysteinyl leukotriene receptor type 1 (Anderson et al. 2009). Mon is an anti-inflammatory agent approved by the Food and Drug Administration for the treatment of various inflammatory conditions such as asthma and rheumatoid arthritis. The safety profile of Mon with daily and chronic use has been well-reported over the past two decades (Okunishi and Peters-Golden 2011; Osher et al. 2006). Recently, it was reported that Mon is effective in preventing diabetic retinopathy by targeting retinal microvascular permeability and inflammation (Bapputty et al. 2019). However, little is known about the impact of Mon on DN. Thus, our study aims at investigating the potential nephroprotective effects of the systemic administration of Mon as a beneficial preventive therapy in experimentally induced DN in rats.

## Materials and methods

### Experimental animals

Adult male Sprague–Dawley (SD) rats weighing between 180 and 200 g were purchased from VACSERA (Agouza, Giza, Egypt). The rats were housed for two weeks with 12 h light/dark cycle and unlimited access to food and water, ad libitum. This study received approval from the Faculty of Pharmacy's Scientific Research Ethics Committee (code number: 2023-41).

### Induction of diabetes

SD rats were rendered diabetic by single I.P injection of STZ (Sigma-Aldrich (St. Louis, MO, USA), 55 mg/kg,) dissolved in a citrate buffer (pH 4.5) after fasting for 8 h (Seedevi et al. 2020). Evaluation of blood glucose was done by examination of samples from tail puncture three days after STZ injection using an Accu-chek go blood glucose meter (Roche Diabetes Care, India). Rats with fasting blood glucose levels (6 h fasting) above 300 mg/dl were considered as diabetic.

### Experimental design

After the induction of diabetes, rats were randomly divided into five groups. 1. Normal control (Ctrl, 6 rats) group; received 0.9% w/v saline (2 ml/kg/day) for eight weeks orally using oral gavage. 2. Diabetes mellitus (DM, 10 rats) group; received STZ and after three days received 2 ml/kg/day 0.9% w/v saline for eight weeks orally using oral gavage. 3. Mon (Sandoz (Cairo, Egypt), 10 mg/kg, 8 rats) group; received STZ and after three days received Mon (10 mg/kg) dissolved in 0.9% w/v saline for eight weeks orally using oral gavage. 4. Mon (20 mg/kg, 8 rats) group; received STZ and after three days received Mon (20 mg/kg) dissolved in 0.9% w/v saline for eight weeks orally using oral gavage. 5. Ins (Mixtard: soluble Ins 30% and isophane Ins 70%), purchased from local pharmacy (Mansoura, Egypt), 8 rats) group; received STZ and after three days received Ins (1.5 IU/animal) for eight weeks by I.P injection. Mon tested doses were selected according to preliminary study and referring to other studies (Otunctemur et al. 2015; Beytur et al. 2012; Khodir et al. 2014), so was the dose for Ins (Luippold et al. 2016; Grant et al. 2012).

8 weeks following STZ injection, fasting blood glucose (6 h fasting) was evaluated by examination of blood samples obtained from tail puncture using an Accu-check go blood glucose meter. Then, rats were anesthetized by I.P injection of thiopental (40 mg/kg), and the blood was collected from retro-orbital venous plexus. Blood samples were centrifuged at 4000 rpm for 15 min at 4 °C and serum was separated. Serum samples were divided into aliquots for determination of serum creatinine, blood urea nitrogen (BUN), malondialdehyde (MDA), and total antioxidant capacity (TAC). The kidneys were cut longitudinally into two portions. One portion was fixed in 10% v/v neutral buffered formalin solution for 24 h and standard histopathological techniques were followed. The other portion was used to prepare (10% w/v) tissue homogenate as described by Bueg and Aust (Buege and Aust 1978) for biochemical assays, as discussed in the following section(s).

### Assessment of kidney function biomarkers; serum creatinine, and BUN

Ready to use assay kits were purchased from biomed (Badr City, Egypt) and used for determination of creatinine and BUN in different serum samples. The steps were carried out in context with the manufacturer's instructions.

### Assessment of serum MDA and TAC

Serum MDA and TAC were evaluated using ready to use assay kits purchased from Biodiagnostic (Giza, Egypt). The steps were carried out in context with the manufacturer's instructions.

### Histopathological examination

10% formalin fixed kidney portions were embedded in paraffin wax blocks with subsequent sectioning into slices, and stained with hematoxylin and eosin (H&E) stain for histological examination (Feldman and Wolfe 2014). Evaluation of the renal injury was semi-quantitively done by a histopathologist in blinded fashion (Magnifications: ×100 bar 100 and ×400 bar 50). Randomly selected 20 fields were quantified. Tubular dilatation, tubular atrophy, tubular vacuolization, deterioration of tubular epithelial cells, epithelial and leukocytic cell casts, and thickening of the tubular basement membrane were considered signs of tubular damage. Renal histopathological score was done using the following scoring system as described by Kuruş et al. (2009) (0: normal; 1: < 10% of the tubules are involved; 2: 10–25%; 3: 26–50%; 4: 51–75%; 5: 76–100%). Quantification of interstitial fibrosis was done by staining the sections with Masson trichrome stain. The fibrotic area was measured in the outer stripe of outer medulla and cortico-medullary junction avoiding the blood vessels (Yamate et al. 1995). (Magnifications: ×100 bar 100 and X400 bar 50).

### Evaluations of renal content of HMGB1, NF-κB, NLRP3, and p62 by western blot

Homogenized renal tissue samples were combined with the ReadyPrepTM protein extraction kit (total protein) provided by Bio-Rad Inc (Cat. # 163-2086) in accordance with the manufacturer's instructions. In order to calculate the protein content in each sample, a Bradford assay was carried out using Bradford Protein Assay Kit (Cat. # SK3041, Bio Basic inc. (Ontario, Canada)), as per the manufacturer's instructions. Equal volumes of the samples (20 ug protein) were mixed with 2 × Laemmli buffer containing 4% sodium dodecyl sulfate (SDS), 10% 2-mercaptoethanol, 20% glycerol, 0.004% bromophenol blue, and 0.125 M Tris HCl, PH 6.8, and each mixture was heated for five minutes at 95 °C for protein denaturation. Protein samples were separated by electrophoresis using SDS-PAGE gel, then blotted into polyvinylidene fluoride membrane. The membrane was blocked for one hour at room temperature in 3% bovine serum albumin prepared in tris-buffered saline with Tween 20 (TBST, 0.1%), then incubated for overnight at 4 °C with the primary antibody for HMGB1 (Cat. # sc-56698), NF-κB (Cat. # sc-8008), and p62 (Cat. # sc-48389) purchased from Santa Cruz Biotechnology, Inc. (Dallas, TX, USA), and incubated for overnight at 4 °C with the primary antibody of NLRP3 (Cat. # ab263899) purchased from Abcam, Waltham, MA, USA. Membranes were then washed with TBST and incubated with the corresponding HRP-conjugated 2nd antibody (Novus Biologicals) for 1 h at room temperature. The blot was washed with TBST and chemiluminescent signal was developed using the chemiluminescent substrate (Cat. # 170-5060, Bio-Rad, USA), as per the manufacturer instructions. The chemiluminescent signals were captured using a CCD camera-based imager. Image analysis software was used to read the band intensity of the target proteins against Ctrl sample beta actin (housekeeping protein) by protein normalization on the ChemiDoc MP imager (Bio-Rad, USA).

### Assessment of renal TLR4, NF-κB (in nucleus), IL1β, NLRP3, p62, and LC3-II

The prepared 10% w/v renal tissue homogenates were centrifuged at 10,000 rpm for 20 min to remove the mitochondria, erythrocytes, unbroken cells, cell debris, and nuclei. The supernatant, comprising cytoplasmic fraction was used for ELISA estimation of TLR4 (Cat. # SEA753Ra) and IL1β (Cat. # SEA563Ra), using commercially available kits from Cloud-Clone Corp., TX, USA, as per the manufacturer instructions. Cytoplasmic fraction was also used for ELISA estimation of NOD-like receptor family pyrin domain containing (NLRP) 3 (Cat. # OKCD04232-48, Aviva Systems Biology (California, USA)), p62 (Cat. # SL1363RA, Sunlong Biotech Co., Ltd (Hangzhou, China)), and LC3-II (Cat. # CBA-5116, Cell Biolabs Inc (California, USA)), as per the manufacturer instructions for commercial kits. The nuclear pellet was resuspended in 100 µl nuclear lysis buffer by pipetting up and down. The suspension was centrifuged at 14,000 rpm for 10 min at 4 °C. The supernatant (Nuclear Fraction) was transferred into a pre-chilled microcentrifuge tube for estimation of nuclear NF-κB (Cat. # NBP2-29661, Novus biologicals (Colorado, United States)). Tissue protein assessment was carried out in context with the method of Bradford et al. (1976) using Genei, Bangalore, and protein estimation kits.

### Immunohistochemistry of LC3-II

After deparaffinization, dehydration, blocking of peroxidase activity, and antigen retrieval, renal sections were incubated with normal rabbit serum to block nonspecific binding of antibodies. Renal sections were then incubated overnight with rabbit polyclonal primary antibodies against LC3-II (ab192890, Abcam). Sections were incubated with secondary antibody-HRP and counterstained with Mayer's hematoxylin.

### Statistical analyses

Data are expressed as mean ± SEM of each group of rats. A one-way analysis of variance (ANOVA) was used for statistical comparisons, followed by the Tukey–Kramer post hoc test. A non-parametric Kruskal–Wallis test was utilized to study the histopathological scores, followed by a post hoc Dunn's multiple comparison tests, and the data are shown as median with interquartile range. Statistical analysis and graphing were performed with the aid of GraphPad Prism Version 8 (GraphPad Software Inc., San Diego, CA, USA).

## Results:

### Effect of Mon administration on fasting blood glucose level, serum creatinine, and BUN in diabetic rats

**Fig. 1:**
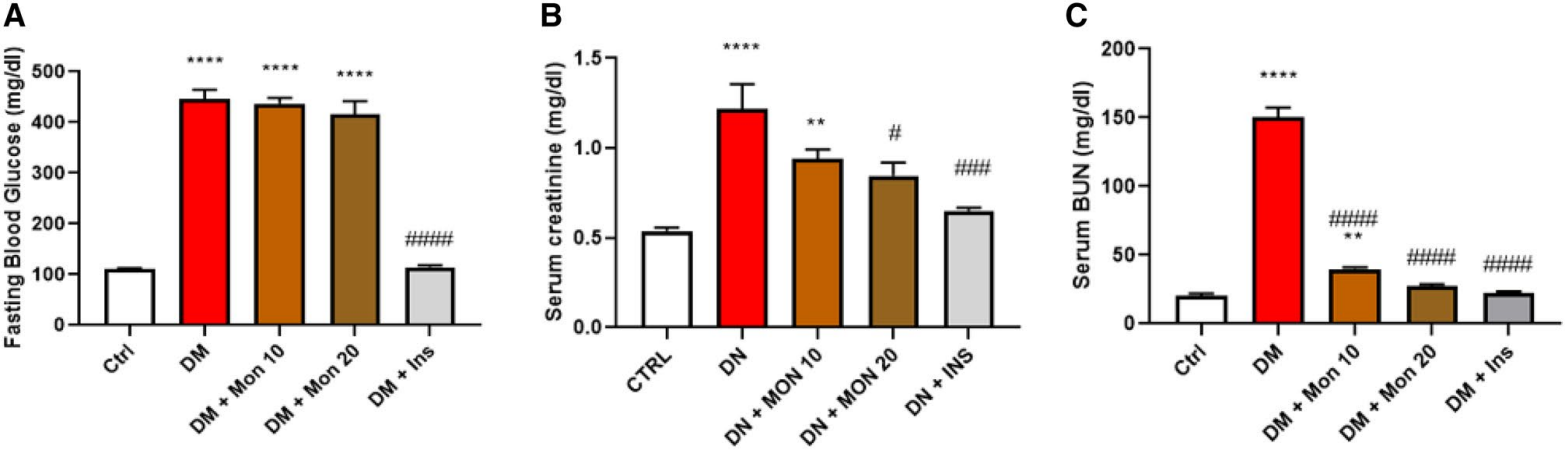
Effect of Mon administration on fasting blood glucose level (**A**), serum creatinine (**B**), and blood urea nitrogen (**C**) in diabetic rats. Diabetes mellitus (DM) was induced in SD rats by single streptozotocin (STZ) injection: Mon (10 and 20 mg/kg) and Ins (1.5 IU/ animal) were administered once daily for 8 weeks, 3 days post STZ injection. Data is represented as mean ± SEM (6 rats /group). DM resulted in significant increase in fasting blood glucose, serum creatinine, and serum BUN, that was attenuated by Mon administration. Ins (1.5 IU/animal) was used as a positive control treatment. One-way analysis of variance (ANOVA) was used to statistically examine the data, and the Tukey–Kramer multiple comparisons test was used as a post-hoc analysis. **, **** Significantly different (*P* < 0.01, 0.0001), respectively, compared to Ctrl group. #, ###, #### Significantly different (*P* < 0.05, 0.001, 0.0001), respectively, compared to DM group

Single I.P. injection of STZ (55 mg/kg) significantly (*P* < 0.0001) increased fasting blood glucose (Fig. 1A), serum creatinine (Fig. 1B) and BUN (Fig. 1C) levels by ~ 4, 2.1, and 7.4 folds, respectively, compared to Ctrl group. Daily treatment with Mon 10 and 20 mg/kg failed to significantly reduce fasting blood glucose compared to DM group (Fig. 1A). Mon (10 mg /kg) reduced serum creatinine by ~ 23%, that was not significant compared to DM group, whereas Mon (20 mg/kg) significantly (*P* < 0.05) reduced serum creatinine by ~ 30.7% compared to DM group to reach a comparable level to that in Ctrl group (Fig. 1B). Meanwhile, the two doses significantly (*P* < 0.0001) decreased BUN by 70.5% and 81.9%, respectively, compared to DM group with Mon (20 mg/kg) group reaching comparable levels to that of Ctrl group (Fig. 1C). Ins (1.5 IU/animal) significantly reduced fasting blood glucose (Fig. 1A), serum creatinine (Fig. 1B) and BUN (Fig. 1C) levels by ~ 74.2% (*P* < 0.0001), 46.7% (*P* < 0. 001), and 84.9% (*P* < 0.0001), respectively, compared to DM group reaching a comparable level to that of Ctrl group.

### Effect of Mon administration on serum MDA content, and TAC in diabetic rats

**Fig. 2:**
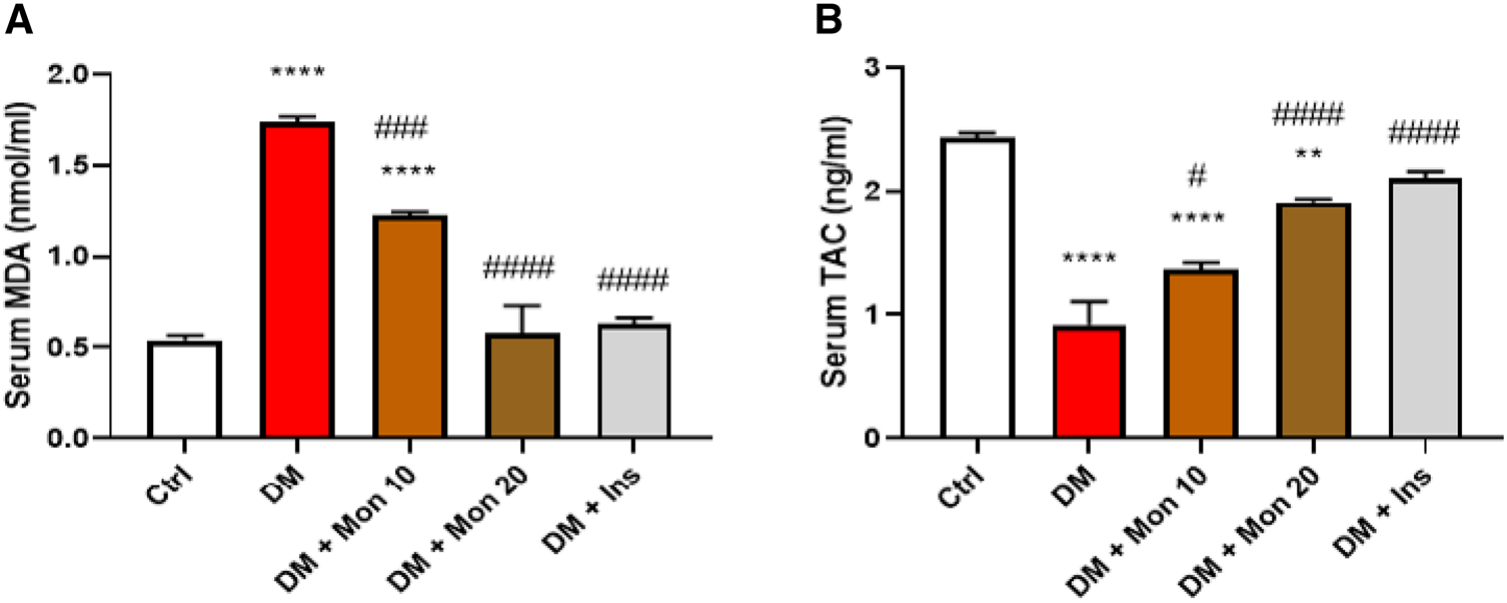
Effect of Mon administration on serum malondialdehyde (**A**) and total antioxidant capacity (**B**) in diabetic rats. Diabetes mellitus (DM) was induced in SD rats by single streptozotocin (STZ) injection: Mon (10 and 20 mg/kg) and Ins (1.5 IU/ animal) were administered once daily for 8 weeks, 3 days post STZ injection. Values are expressed as mean ± SEM (n = 6/ group). DM resulted in significant increase in serum MDA and TAC, that was attenuated by Mon administration. Ins (1.5 IU/animal) was used as a positive control treatment. One-way analysis of variance (ANOVA) was used to statistically examine the data, and the Tukey–Kramer multiple comparisons test was used as a post-hoc analysis. **, **** Significantly different (*P* < 0.01, 0.0001), respectively, compared to Ctrl group. #, ###, #### Significantly different (*P* < 0.05, 0.001, 0.0001), respectively, compared to DM group

Kidney oxidants/antioxidants balance was significantly dysregulated in DM group as evidenced by the significant (*P* < 0.0001) increase in MDA content by ~ 3.2 folds (Fig. 2A), and the significant (*P* < 0.0001) decrease in TAC by 62.7% compared to the Ctrl group (Fig. 2B). Mon (10 and 20 mg/kg) significantly (*P* < 0.001*,* 0.0001) reduced MDA by ~ 28.2%, 51.2%, respectively, compared to DM group (Fig. 2A). Meanwhile, the two doses significantly increased TAC by 1.5 (*P* < 0.05) and 2.1 (*P* < 0.0001) folds, respectively, compared to DM group (Fig. 2B). Ins (1.5 IU/animal) significantly (*P* < 0.0001) reduced MDA by 63.2% (Fig. 2A), and (*P* < 0.0001) increased TAC by 2.3-fold (Fig. 2B) compared to DM group and to reach a comparable level of its oxidants/antioxidants balance to that of Ctrl group.

### Effect of Mon administration on renal histopathological changes in diabetic rats

**Fig. 3:**
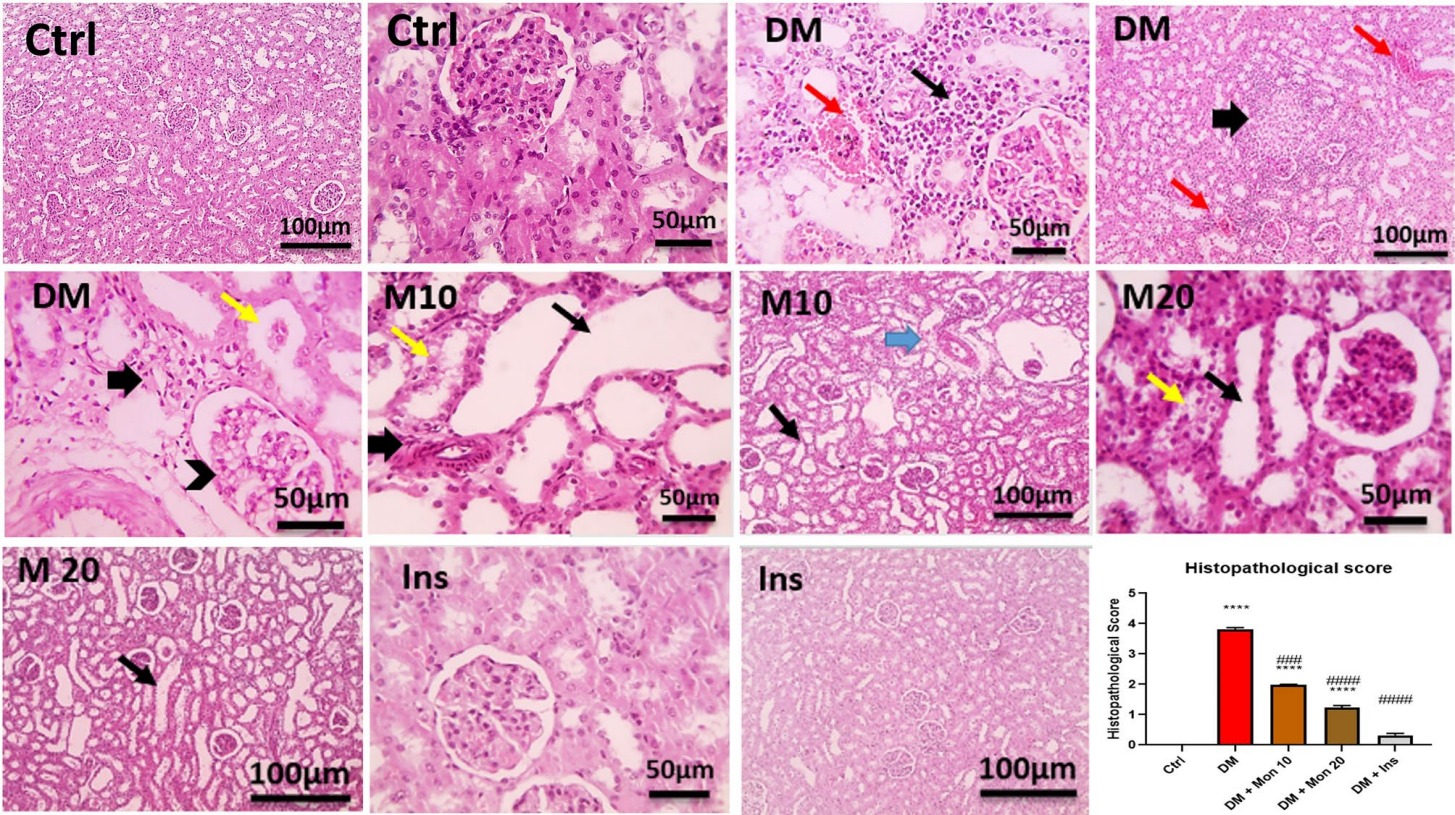
Effect of Mon administration on renal histopathological changes in diabetic rats. Diabetes mellitus (DM) was induced in SD rats by single streptozotocin (STZ) injection: Mon (10 and 20 mg/kg) and Ins (1.5 IU/ animal) were administered once daily for 8 weeks, 3 days post STZ injection. Values are expressed as median with interquartile range. DM resulted in a significant increase in renal injury, that was decreased by Mon administration. Ins (1.5 IU/animal) was used as a positive control treatment. Data were statistically analyzed using Kruskal–Wallis test followed by Dunn's multiple comparison post-hoc test. **** Significantly different (*P* < 0.0001) compared to Ctrl group. ###, #### Significantly different (*P* < 0.001, 0.0001), respectively, compared to DM group. In DM group, yellow arrows represent epithelial and leukocytic cells casts, thick black arrows indicate focal fibrosis, thin black arrows represent mononuclear cell infiltration in interstitial tissue, and red arrow represent congested blood vessels, and arrowhead represent widened Bowman's space with slightly thickened glomerular basement membrane. In Mon groups, thin black arrows represent dilated tubules with flattened lining epithelium that lost their brush borders, yellow arrows indicate mild hydropic degeneration, thick black arrows represent focal interstitial fibrosis, and thick blue arrows indicate mild perivascular edema. Low magnification X: 100 bar 100 and high magnification X: 400 bar 50

Examination of renal sections stained by H&E from the Ctrl group showed normal glomeruli and tubules with minimal interstitial tissue. In contrast, renal sections from DM group showed tubular dilation, epithelial and leukocytic cells casts, focal fibrosis, mononuclear cell infiltration in interstitial tissue, and congested blood vessels. Widened Bowman's space with slightly thickened glomerular basement membrane was seen in some sections. Renal sections from group Mon (10 mg/kg) showed dilated tubules with flattened lining epithelium that lost their brush borders, and mild hydropic degeneration. Focal interstitial fibrosis and mild perivascular edema were noticed in some sections. Renal sections from group Mon (20 mg/kg) showed dilated tubules with flattened lining epithelium that lost their brush border, and mild hydropic degeneration in a few tubules. Renal sections from the Ins group showed normalized pictures of glomeruli and tubules (Fig. 3). The scoring of the renal injury indicated that single I.P. injection of STZ (55 mg/kg) significantly (*P* < 0.0001) increased renal injury compared to Ctrl group. Mon (10 and 20 mg/kg) significantly (*P* < 0.001, 0.0001), respectively, reduced renal injury score compared to DM group. Ins (1.5 IU/animal) significantly (*P* < 0.0001) reduced renal injury score compared to DM group and reached a comparable score of that in Ctrl group (Fig. 3).

### Effect of Mon administration on renal fibrosis in diabetic rats

**Fig. 4:**
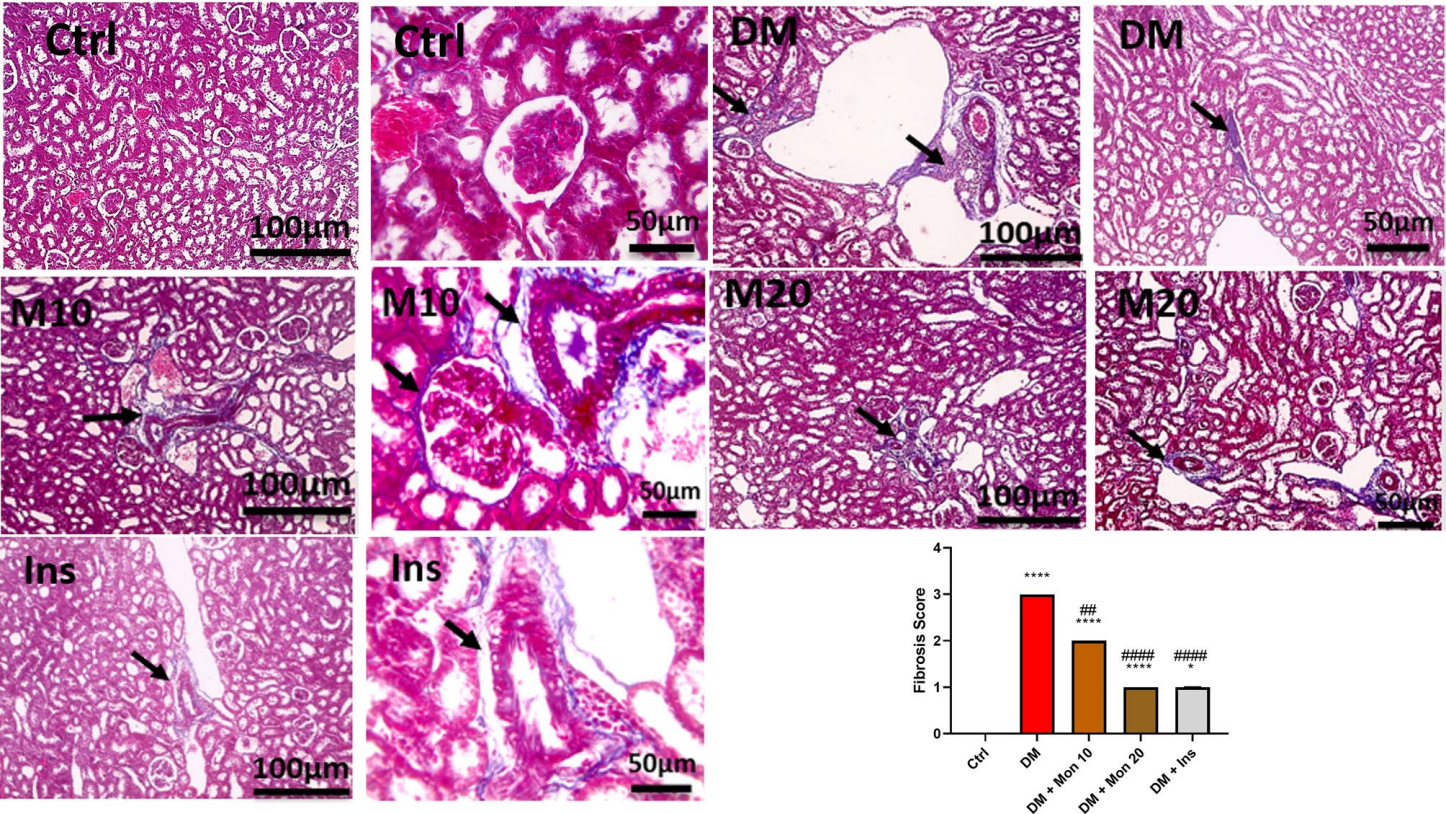
Effect of Mon administration on renal fibrosis in diabetic rats. Diabetes mellitus (DM) was induced in SD rats by single streptozotocin (STZ) injection: Mon (10 and 20 mg/kg) and Ins (1.5 IU/ animal) were administered once daily for 8 weeks, 3 days post STZ injection. Values are expressed as median with interquartile range. DM resulted in significant increase in renal fibrosis, that was decreased by Mon administration. Ins (1.5 IU/animal) was used as a positive control treatment. Data were statistically analyzed using Kruskal–Wallis test followed by Dunn's multiple comparison post-hoc test. *, **** Significantly different (*P* < 0.05, 0.0001), respectively, compared to Ctrl group. ##, #### Significantly different (*P* < 0.01, 0.0001), respectively, compared to DM group. Thin black arrows represent collagen deposition. Low magnification X: 100 bar 100 and high magnification X: 400 bar 50

Microscopic pictures of Masson trichrome stained renal sections showed no fibrosis in the Ctrl group. In contrast, renal sections from DM group showed marked bluish collagen deposition in the interstitial tissue. The collagen deposition decreased in a dose-dependent manner in Mon (10 and 20 mg/kg) groups. The highest decrease in collagen deposition in interstitial tissue was reported in the Ins group (Fig. 4). The scoring of the renal fibrosis indicated that single I.P. injection of STZ (55 mg/kg) significantly (*P* < 0.0001) increased renal fibrosis compared to Ctrl group. Mon (10 and 20 mg/kg) significantly (*P* < 0.01, 0.0001), respectively, reduced renal fibrosis score compared to DM group. Ins (1.5 IU/animal) significantly (*P* < 0.0001) reduced renal fibrosis score compared to DM group (Fig. 4).

### Effect of Mon administration on renal expression of HMGB1 and TLR4 in diabetic rats

**Fig. 5:**
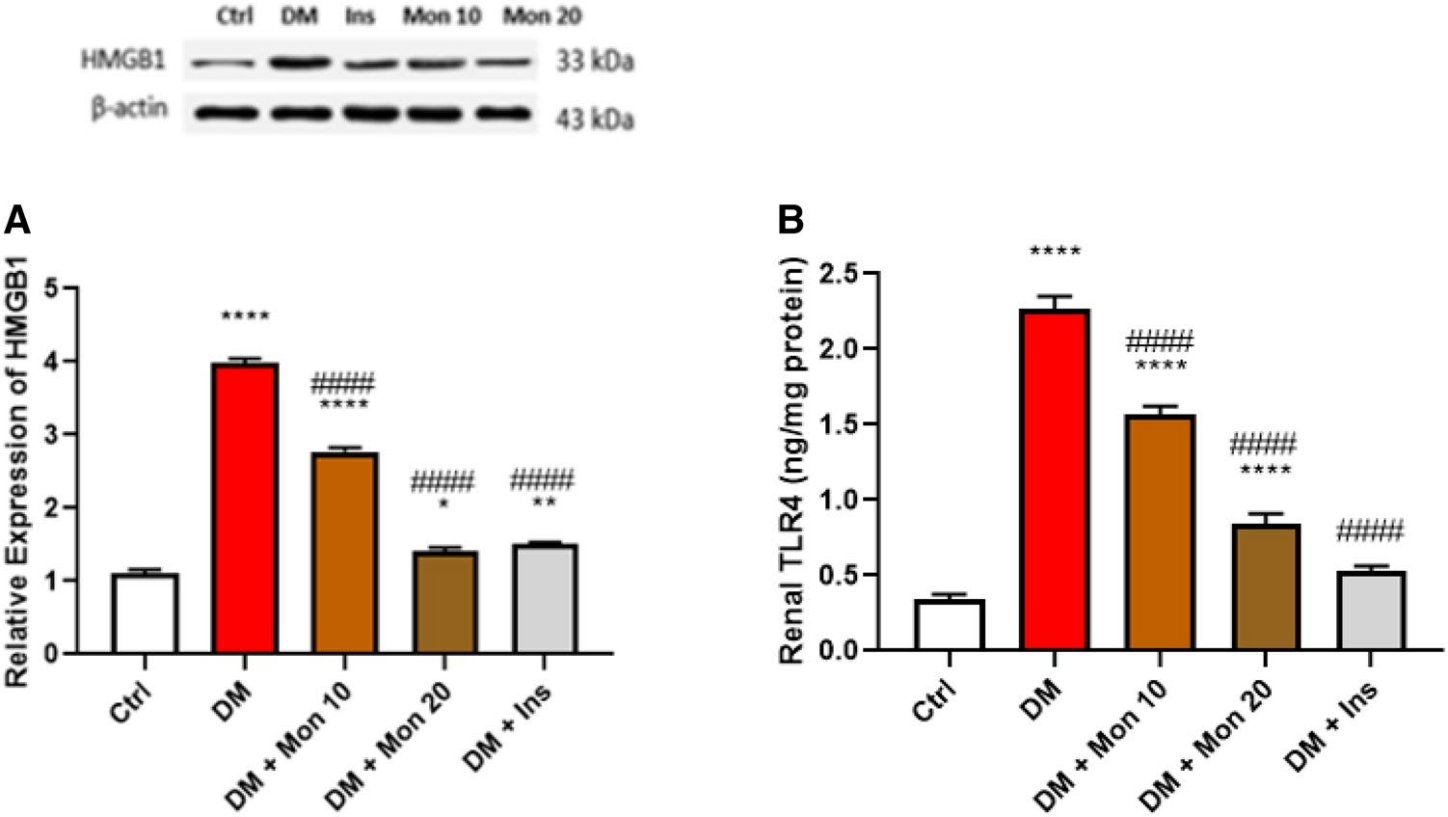
Effect of Mon administration on the renal expression of high mobility group box 1 (**A**) and toll like receptor 4 (**B**) in diabetic rats. Diabetes mellitus (DM) was induced in SD rats by single streptozotocin (STZ) injection: Mon (10 and 20 mg/kg) and Ins (1.5 IU/ animal) were administered once daily for 8 weeks, 3 days post STZ injection. Values are expressed as mean ± SEM (n = 6/group). DM resulted in significant increase in renal expression of HMGB1 and TLR4, that was decreased by Mon administration. Ins (1.5 IU/animal) was used as a positive control treatment. One-way analysis of variance (ANOVA) was used to statistically examine the data, and the Tukey–Kramer multiple comparisons test was used as a post-hoc analysis. *, **, **** Significantly different (*P* < 0.05, 0.01, 0.0001), respectively, compared to Ctrl group. #### Significantly different (*P* < 0.0001) compared to DM group

Single I.P. injection of STZ (55 mg/kg) significantly (*P* < 0.0001) increased the expression of HMGB1 (Fig. 5A), and TLR4 (Fig. 5B) by ~ 3.9 and 6.7 folds, respectively, compared to the Ctrl group. Mon (10 mg/kg) significantly (*P* < 0.0001) reduced both HMGB1 (Fig. 5A) and TLR4 (Fig. 5B) by ~ 31.4%, and 30.7%, respectively, compared to DM group. Mon (20 mg/kg) significantly (*P* < 0.0001) reduced both HMGB1 (Fig. 5A) and TLR4 (Fig. 5B) by ~ 66.6%, and 62.9%, respectively, compared to DM group. Ins (1.5 IU/animal) significantly (*P* < 0.0001) reduced both HMGB1 (Fig. 5A) and TLR4 (Fig. 5B) by ~ 62.2%, and 76.8%, respectively, compared to DM group, rendering TLR4 level compared to that of the Ctrl group.

### Effect of Mon administration on renal expression of NF-κB (in the nucleus), IL-1β, and NLRP3 in diabetic rats

**Fig. 6:**
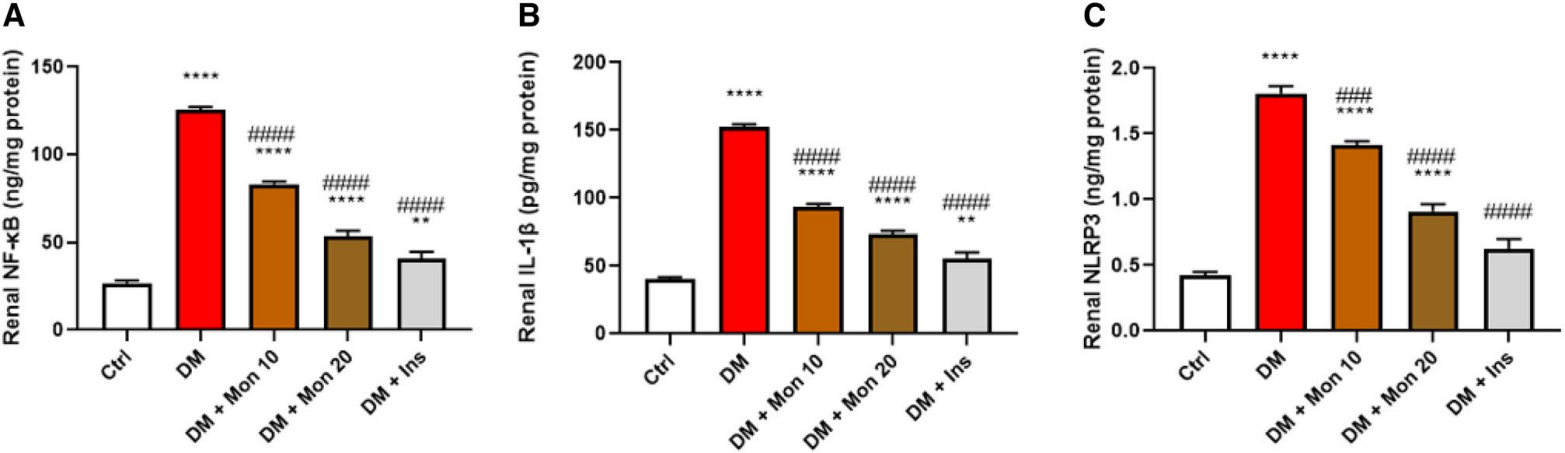
Effect of Mon administration on the renal expression of nuclear factor kappa B (in nucleus) (**A**), interleukin -1β (**B**), and NOD-like receptor family pyrin domain containing 3 (**C**) in diabetic rats. Diabetes mellitus (DM) was induced in SD rats by single streptozotocin (STZ) injection: Mon (10 and 20 mg/kg) and Ins (1.5 IU/ animal) were administered once daily for 8 weeks, 3 days post STZ injection. Values are expressed as mean ± SEM (n = 6/ group). DM resulted in significant increase in renal expression of NF-κB (in nucleus), NLRP3, and IL -1β, that was decreased by Mon administration. Ins (1.5 IU/animal) was used as a positive control treatment. One-way analysis of variance (ANOVA) was used to statistically examine the data, and the Tukey–Kramer multiple comparisons test was used as a post-hoc analysis. **, **** Significantly different (*P* < 0.01, 0.0001), respectively, compared to Ctrl group. ###, #### Significantly different (*P* < 0.001, 0.0001), respectively, compared to DM group

Single I.P. injection of STZ (55 mg/kg) significantly (*P* < 0.0001) increased renal expression of NF-κB (in the nucleus) (Fig. 6A), IL-1β (Fig. 6B), and NLRP3 (Fig. 6C) by ~ 4.6, 3.8, and 4.3 folds, respectively, compared to the Ctrl group. Mon (10 mg/kg) significantly reduced renal expression of NF-κB (Fig. 6A), IL-1β (Fig. 6B), and NLRP3 (Fig. 6C) by ~ 33.7% (*P* < 0.0001), 38.8% (*P* < 0.0001), and 22.2% (*P* < 0. 001), respectively, compared to DM group. Mon (20 mg/kg) significantly (*P* < 0.0001) reduced renal expression NF-κB (Fig. 6A), IL-1β (Fig. 6B), and NLRP3 (Fig. 6C) by ~ 57.3%, 51.8%, and 49.4%, respectively, compared to DM group. Ins (1.5 IU/animal) significantly (*P* < 0.0001) reduced renal expression of NF-κB (Fig. 6A), IL-1β (Fig. 6B), and NLRP3 (Fig. 6C) by ~ 67.5%, 63.7%, and 65.3%, respectively, compared to DM group. Notably, the administration of Ins decreased renal levels of NLRP3 to Ctrl values.

### Effect of Mon administration on renal expression of p62 and LC3-II in diabetic rats

**Fig. 7:**
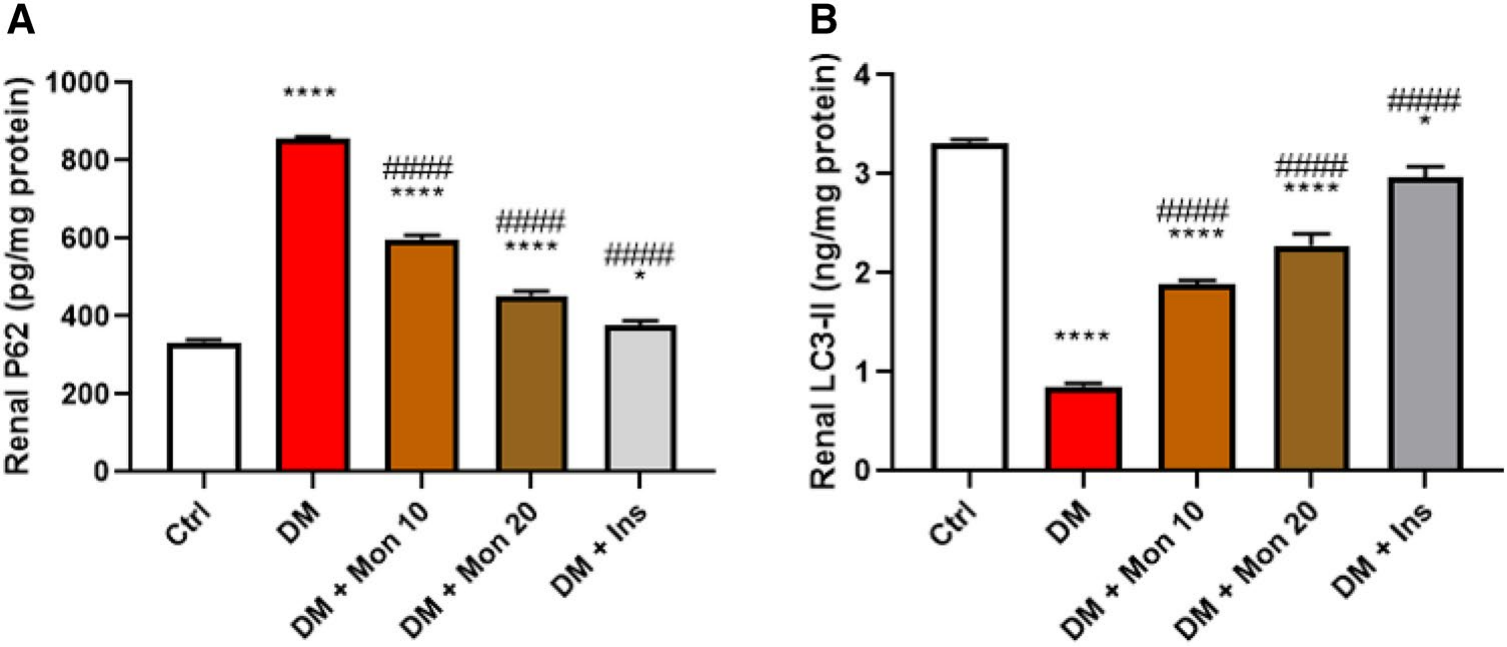
Effect of Mon administration on the renal expression of p62 (**A**) and microtubule-associated protein 1A/1B-light chain 3 -II (**B**) in diabetic rats. Diabetes mellitus (DM) was induced in SD rats by single streptozotocin (STZ) injection: Mon (10 and 20 mg/kg) and Ins (1.5 IU/ animal) were administered once daily for 8 weeks, 3 days post STZ injection. Values are expressed as mean ± SEM (n = 6/ group). DM resulted in significant dysregulation in autophagy, as evidenced by p62 and LC3-II levels, that was decreased by Mon administration. Ins (1.5 IU/animal) was used as a positive control treatment. One-way analysis of variance (ANOVA) was used to statistically examine the data, and the Tukey–Kramer multiple comparisons test was used as a post-hoc analysis. *, **** Significantly different (*P* < 0.05, 0.0001), respectively, compared to Ctrl group. #### Significantly different (*P* < 0.0001) compared to DM group

Autophagy was significantly dysregulated in DM group as evidenced by the marked (*P* < 0.0001) increase in p62 by ~ 2.6 folds (Fig. 7A), and the significant (*P* < 0.0001) decrease in LC3-II by 74.8% (Fig. 7B) compared to the Ctrl group. Mon (10 and 20 mg/kg) significantly (*P* < 0.0001) reduced p62 level by ~ 30.1%, 47%, respectively, compared to DM group (Fig. 7A). Meanwhile, the two doses of Mon; 10 & 20 mg/kg significantly (*P* < 0.0001) increased LC3-II level by ~ 2.2 and 2.7 folds, respectively, compared to DM group (Fig. 7B). Ins (1.5 IU/animal) significantly (*P* < 0.0001) reduced p62 by 55.8% (Fig. 7A), and (*P* < 0.0001) increased LC3-II by ~ 3.5 fold (Fig. 7B) compared to DM group.

### Effect of Mon administration on renal levels of NF-κB, NLRP3, and p62, measured by western blot, in diabetic rats

**Fig. 8:**
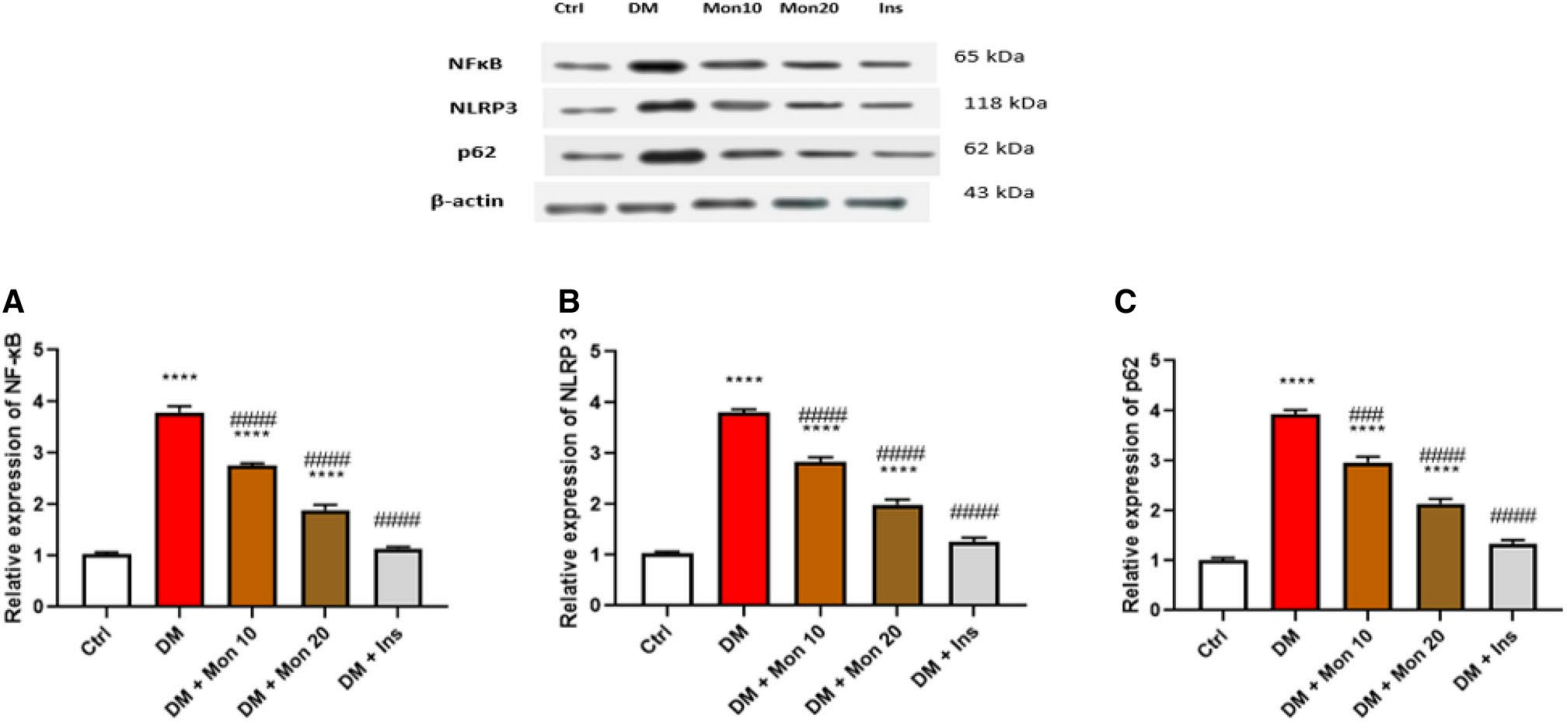
Effect of Mon administration on the renal levels of nuclear factor kappa B (**A**), NOD-like receptor family pyrin domain containing 3 (**B**), and p62 (**C**), measured by western blot, in diabetic rats. Diabetes mellitus (DM) was induced in SD rats by single streptozotocin (STZ) injection: Mon (10 and 20 mg/kg) and Ins (1.5 IU/ animal) were administered once daily for 8 weeks, 3 days post STZ injection. Values are expressed as mean ± SEM. DM resulted in significant increase in renal expression of NF-κB, NLRP3, and p62, that was decreased by Mon administration. Ins (1.5 IU/animal) was used as a positive control treatment. One-way analysis of variance (ANOVA) was used to statistically examine the data, and the Tukey–Kramer multiple comparisons test was used as a post-hoc analysis. **** Significantly different (*P* < 0.0001) compared to Ctrl group. ###, #### Significantly different (*P* < 0.001, 0.0001), respectively, compared to DM group

In addition to ELISA, these markers were also measured by western blot to further validate the effect of Mon on inflammation and autophagy. Single I.P. injection of STZ (55 mg/kg) significantly (*P* < 0.0001) increased renal levels of NF-κB (Fig. 8A), NLRP3 (Fig. 8B), and p62 (Fig. 8C) compared to Ctrl group. Mon (10 mg/kg) significantly (*P* < 0.0001) decreased renal levels of NF-κB (Fig. 8A), NLRP3 (Fig. 8B), and significantly (*P* < 0.001) decreased renal levels of p62 (Fig. 8C) compared to DM group. Mon (20 mg/kg) significantly (*P* < 0.0001) decreased renal levels of NF-κB (Fig. 8A), NLRP3 (Fig. 8B), and p62 (Fig. 8C) compared to DM. Ins (1.5 IU/animal) significantly (*P* < 0.0001) decreased renal levels of NF-κB (Fig. 8A), NLRP3 (Fig. 8B), and p62 (Fig. 8C) compared to DM, and reached comparable levels of the 3 markers to that seen in Ctrl group.

### Effect of Mon administration on renal levels of LC3-II, measured by immunohistochemistry, in diabetic rats

**Fig. 9:**
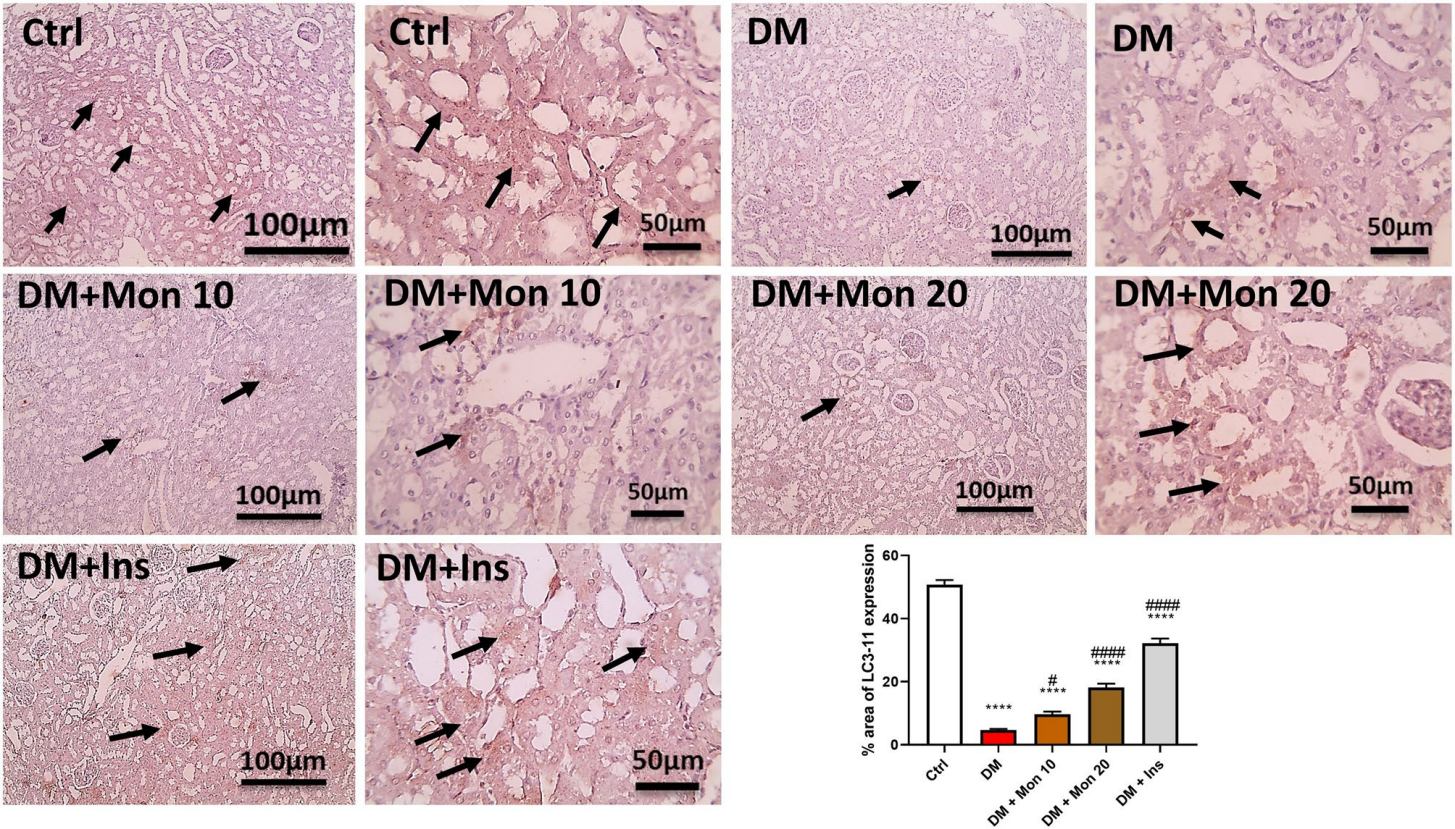
Effect of Mon administration on the renal level of microtubule-associated protein 1A/1B-light chain 3 -II, measured by immunohistochemistry, in diabetic rats. Diabetes mellitus (DM) was induced in SD rats by single streptozotocin (STZ) injection: Mon (10 and 20 mg/kg) and Ins (1.5 IU/ animal) were administered once daily for 8 weeks, 3 days post STZ injection. Values are expressed as mean ± SEM. DM resulted in significant increase in renal expression of LC3-II, that was decreased by Mon administration. Ins (1.5 IU/animal) was used as a positive control treatment. One-way analysis of variance (ANOVA) was used to statistically examine the data, and the Tukey–Kramer multiple comparisons test was used as a post-hoc analysis. **** Significantly different (*P* < 0.0001), respectively, compared to Ctrl group. #, #### Significantly different (*P* < 0.05, 0.0001), respectively, compared to DM group. Thin black arrow represents brown tubular reaction of LC3-II content.. Low magnification X: 100 bar 100 and high magnification X: 400 bar 50

In addition to ELISA, LC3-II was also measured by immunohistochemistry to further validate the effect of Mon on autophagy. Autophagy was significantly dysregulated in DM group as evidenced by the significant (*P* < 0.0001) decrease in LC3-II (Fig. 9) compared to the Ctrl group. Mon (10 mg/kg) significantly (*P* < 0.05) increased LC3-II level compared to DM group, and Mon (20 mg/kg) significantly (*P* < 0.0001) increased LC3-II level compared to DM group (Fig. 9). Ins (1.5 IU/animal) significantly (*P* < 0.0001) increased LC3-II compared to DM group (Fig. 9). The microscopic pictures of immunostained renal sections against LC3-II showing prominent positive brown tubular reaction in Ctrl group. Meanwhile, renal sections from DM group showing markedly decreased positive brown tubular reaction against LC3-II. Renal sections from Mon 10 mg/kg group showing slightly increased positive brown tubular reaction against LC3-II. Renal sections from Mon 20 mg/kg group showing moderately increased positive brown tubular reaction against LC3-II. Renal sections from Ins (1.5 IU/animal) group showing the higher increase positive brown tubular reaction against LC3-II.

## Discussion

To the best of our knowledge, our study provides the first evidence for Mon in protecting against kidney damage in an STZ-induced model of DN. We show that the nephroprotective impact of Mon against STZ-induced DN in rats involves novel cell signaling pathways, including HMGB1, TLR4, NLRP3, and autophagy. Interestingly, unlike Ins provided as a standard treatment for hyperglycemia, Mon treatment was associated with significant functional, biochemical, and histopathological improvements of the injured kidneys but with no effect on systemic hyperglycemia in the rats.

It was reported that all four renal components, including the glomerulus, interstitial compartment, tubules, and blood vessels, are influenced by diabetes (Parving et al. 1981; Abbate et al. 2006). DN is presented with albuminuria and increased serum creatinine, and BUN levels (Parving et al. 1981; Abbate et al. 2006). Accordingly, serum creatinine and BUN determination can be considered as a good diagnostic marker to detect even minor deterioration in renal function (Awad et al. 2020; Saleh et al. 2020). In addition, glomerular histopathological features in chronic kidney diseases include the thickening of both glomerular basement membrane, mesangial expansion, glomerular sclerosis, and effacement of podocytes (Higgins and Coughlan 2014; Saleh et al. 2020; Awad et al. 2020). In the present study, we show a significant elevation in serum creatinine and BUN in diabetic rats that correlated with tubular dilation, epithelial and leukocytic cell casts, focal fibrosis, mononuclear cell infiltration of interstitial tissue, and congested blood vessels with the slightly thickened glomerular basement membrane. Interestingly, renal sections from diabetic rats that received Mon decreased all the parameters in a dose-dependent manner, suggesting that Mon imparts protection directly to the kidney glomerulus. Indeed, this effect of Mon is consistent with its protective effects in other models of kidney diseases (Suddek 2013, Khodir et al. 2014).

HMGB1, discovered over 30 years ago, is a highly conserved non-histone DNA binding protein, which is widely distributed among various organs, such as lung, brain, liver, heart, and kidney, and it can be released from necrotic cells through active secretion and passive release, inducing inflammation (Goodwin et al. 1973). Under normal circumstances, it participates in many biological processes, such as transcription, DNA repair, and differentiation (Tang et al. 2011). However, in response to appropriate stimuli, it is released from the nucleus into the extracellular medium, a process in which the inflammasome plays a key role. HMGB1 is released from necrotic or inflammatory cells, including dendritic cells and monocyte/macrophages, as a potent proinflammatory cytokine, and acts through multiple cell-surface receptors including RAGE, TLR2, and TLR4 (Bonaldi et al. 2003). So, HMGB1 is considered as one of the endogenous ligands of TLR4, and the interaction of HMGB1 with TLR4 results in a subsequent translocation of NF-κB from the cytoplasm into the nucleus inducing an inflammatory response (Park et al. 2006). In accordance with these studies, HMGB1 plays central role in the pathogenesis of kidney diseases, including DN. It was reported that hyperglycemia-induced HMGB1 release could induce and stimulate renal injury in diabetic rats. This HMGB1 pathogenic role might be dependent on TLR4 with subsequent Activation of NF-κB. So, HMGB1/TLR4/NF-κB is an important inflammatory signal pathway in renal disorders (Bruchfeld et al. 2011; Rabadi et al. 2012; Leelahavanichkul et al. 2011; Kim et al. 2011). Our current study shows a similar increase in TLR4, HMGB1, and NF-kB in DN. Interestingly, our study provides the first evidence that Mon treatment could ameliorate the elevated levels of HMGB1 and TLR4 in diabetic rats underscoring the potential renoprotective effect of the Mon via decreasing inflammation in DN. Consistent with our results, previous studies utilizing Mon showed protective potential in acute coronary syndrome via down-regulating HMGB1 production in platelets (Alomair et al. 2022).

NF-κB pathway is reported as one of the major inflammatory pathways playing a central role in the pathogenesis of DN. It is usually present in an inactive state inside the cells. Upon activation, NF-κB translocates into the nucleus and increases transcription activity stimulating the expression of adhesion molecules, chemokines, and inflammatory cytokines related to inflammation, all of which are involved in the induction and progression of DN (Mezzano et al. 2004). For example, NF-κB is a key mediator of the priming signal responsible for the activation of NLRP3 inflammasome by stimulating the expression of both NLRP3 and pro-IL-1β, which is then converted to IL-1β by caspase 1 to mediate inflammation (Sun 2011; Sutterwala et al. 2014). Accordingly, NF-κB /NLRP3/ IL-1β axis is a central pathway in DN. Consistent with all the previous studies, in the current study, we show an increased expression of nuclear NF-κB, NLRP3, and IL-1β in diabetic group. However, this expression was decreased by Mon in a dose-dependent manner, an effect that indicates the potential impact of Mon in the treatment of DN. In line with these results, an improvement in oxidants/antioxidant balance, as evidenced by MDA, and TAC as well as histopathological findings is consistent with the effect of targeting NF-κB /NLRP3/ IL-1β pathway in the improvement of chronic renal diseases (Zhang and Wang 2019). In support of our observations, a recent study reported that Mon could meliorate hepatotoxicity by targeting NLRP3 /IL-1β inflammasome pathway (El-Kashef and Zaghloul 2022).

Autophagy, a homeostatic mechanism, is known to be dysregulated in the renal glomerulus and tubules in diabetic conditions, leading to the exacerbation of organelle dysfunction and DN (Yamahara et al. 2013). Furthermore, autophagy can influence the development of inflammatory cells i.e., macrophages, lymphocytes, and neutrophils, suggesting that modulation of autophagy may result in therapeutic approaches for inflammatory disorders, including diabetes (Matsuzawa-Ishimoto et al. 2018)**.** Cellular autophagy was shown to be reduced in the renal tubules and cortex in DN induced by STZ, with associated renal hypertrophy, renal accumulation of p62, an observation that was reversed by Ins treatment (Han et al. 1997). Much similar to Ins treatment, treatment of diabetic rats with Mon in a dose-dependent manner was able to stimulate autophagy as evidenced by decreased levels of p62 and increased levels of LC3-II. Interestingly, there is a strong interplay between autophagy, ROS and NLRP3 in DN as autophagy induction can degrade AGEs and regulate M1/M2 macrophages with subsequent reduction in inflammation and fibrosis in DN (Zhao et al. 2017). It was also found that autophagy stimulation was associated with the degradation of NLRP3 leading to alleviation of inflammation as well as renal interstitial fibrosis. Based on these findings, there is a complex interaction among ROS, NLRP3 inflammasome, and autophagy in the development of renal fibrosis (Hu et al. 2018). Future studies must explore the potential link of Mon effects on autophagy with NLRP3, ROS, IL-1β, and improvement in renal fibrosis to understand its renoprotective impact against DN better.

We readily recognize a few limitations of our study. Firstly, we have not studied the combination of Ins and Mon and its impact on lowering the dose frequency of Ins in the management of diabetic complications to improve patient compliance with repeated Ins treatment. Secondly, we have not attempted to study the causal connection between Mon and HMGB1 or NRLP3, which can be analyzed using HMGB1 and NLRP3 knockout rodents to confirm its mechanisms in DN. Lastly, we have not studied the cognate receptor-mediated effects of Mon in DN rats. Mon is a well-established leukotriene receptor antagonist. Future studies need to establish if Mon acts via such receptor-mediated mechanism in DN rats.

In summary, the outcomes of this study focuses on the anti-inflammatory and anti-oxidative stress properties of Mon in the kidneys of STZ-induced diabetic rats by representing Mon values in down-regulating renal levels of principal pro-inflammatory mediator HMGB1 and its correspondent receptor TLR-4; the consequences that together produce improvement renal function as evident by the decreased levels of serum BUN and creatinine. It should be highlighted that Mon-related alleviation in oxidative stress accumulation down-regulates the activities of the master pro-inflammatory NF-kB in renal cells with subsequent reduction in NLRP3 and IL-1β. Moreover, the reduced activities of NF-kB lead to decreased expressions of TLR-4 and therefore the inflammatory processes are further suppressed by a positive feedback mechanism. In addition, Mon has autophagy stimulating properties, as seen by the marked decrease in kidney p62 and the increase in LC3-II levels. Based on our pre-clinical studies using STZ-induced diabetic rats, Mon could be proposed to attenuate DN in high-risk individuals; however, further clinical studies are required to establish the utility of Mon.

## Data Availability

Data will be available on reasonable request from the corresponding author.
